# Programmed cell death 4 loss increases tumor cell invasion and is regulated by miR-21 in oral squamous cell carcinoma

**DOI:** 10.1186/1476-4598-9-238

**Published:** 2010-09-10

**Authors:** Patricia P Reis, Miranda Tomenson, Nilva K Cervigne, Jerry Machado, Igor Jurisica, Melania Pintilie, Mahadeo A Sukhai, Bayardo Perez-Ordonez, Reidar Grénman, Ralph W Gilbert, Patrick J Gullane, Jonathan C Irish, Suzanne Kamel-Reid

**Affiliations:** 1 Division of Applied Molecular Oncology, Princess Margaret Hospital, Ontario Cancer Institute, University Health Network, Toronto, Ontario, Canada; 2 Department of Medical Biophysics, University of Toronto, Toronto, Ontario, Canada; 3 Department of Laboratory Medicine and Pathobiology, University of Toronto, Toronto, Ontario, Canada; 4 Department of Computer Science, University of Toronto, Toronto, Ontario, Canada; 5 Ontario Cancer Institute and the Campbell Family Institute for Cancer Research, University Health Network, Toronto, Ontario, Canada; 6 Dalla Lana School of Public Health Sciences, University of Toronto, Toronto, Ontario, Canada; 7 Department of Biostatistics, Princess Margaret Hospital, University Health Network, Toronto, Ontario, Canada; 8 Division Of Cancer Genomics and Proteomics, Ontario Cancer Institute, University Health Network, Toronto, Ontario, Canada; 9 Department of Pathology, Toronto General Hospital, Ontario Cancer Institute, University Health Network, Toronto, Ontario, Canada; 10 Department of Otorhinolaryngology/Head and Neck Surgery, Turku University Central Hospital, Turku, Finland; 11 Department of Biochemistry, Turku University Central Hospital, Turku, Finland; 12 Department of Otolaryngology/Surgical Oncology, Princess Margaret Hospital, Ontario Cancer Institute and The University Health Network, Toronto, Ontario, Canada

## Abstract

**Background:**

The tumor suppressor Programmed Cell Death 4 (*PDCD4*) has been found to be under-expressed in several cancers and associated with disease progression and metastasis. There are no current studies characterizing PDCD4 expression and its clinical relevance in Oral Squamous Cell Carcinoma (OSCC). Since nodal metastasis is a major prognostic factor in OSCC, we focused on determining whether PDCD4 under-expression was associated with patient nodal status and had functional relevance in OSCC invasion. We also examined *PDCD4 *regulation by microRNA 21 (miR-21) in OSCC.

**Results:**

*PDCD4 *mRNA expression levels were assessed in 50 OSCCs and 25 normal oral tissues. *PDCD4 *was under-expressed in 43/50 (86%) OSCCs, with significantly reduced mRNA levels in patients with nodal metastasis (*p = 0.0027*), and marginally associated with T3-T4 tumor stage (*p = 0.054*). PDCD4 protein expression was assessed, by immunohistochemistry (IHC), in 28/50 OSCCs and adjacent normal tissues; PDCD4 protein was absent/under-expressed in 25/28 (89%) OSCCs, and marginally associated with nodal metastasis (*p = 0.059*). A matrigel invasion assay showed that PDCD4 expression suppressed invasion, and siRNA-mediated PDCD4 loss was associated with increased invasive potential of oral carcinoma cells. Furthermore, we showed that miR-21 levels were increased in PDCD4-negative tumors, and that *PDCD4 *expression may be down-regulated in OSCC by direct binding of miR-21 to the 3'UTR *PDCD4 *mRNA.

**Conclusions:**

Our data show an association between the loss of PDCD4 expression, tumorigenesis and invasion in OSCC, and also identify a mechanism of PDCD4 down-regulation by microRNA-21 in oral carcinoma. PDCD4 association with nodal metastasis and invasion suggests that PDCD4 may be a clinically relevant biomarker with prognostic value in OSCC.

## Introduction

Oral squamous cell carcinomas (OSCCs) are malignant oral cavity tumors that account for 24% of all head and neck cancers [1]. The presence of lymph node metastasis (regional disease) affects more than 50% of OSCC patients and it is one of the most important prognostic indicators associated with poor patient survival [2,3]. The probability of distant metastases increases when there is cervical node involvement and survival rates decrease by approximately 50% [4]. Detection of nodal metastasis is important at diagnosis; clinical staging of neck nodes is determined by physical examination of enlarged lymph nodes and imaging. However, even when no nodal involvement is detected, there is still a high incidence (> 20%) of occult neck metastasis [3]. These factors contribute to the high morbidity and mortality rates of patients with OSCC. Based on the hypothesis that metastatic potential may be determined by the genetic properties of the primary tumor [5], studies focused on the ability of biomarkers to predict metastatic potential are urgently needed. Such studies may impact management of neck disease, patient treatment and survival.

Recently, Programmed Cell Death 4 (*PDCD4*) has been strongly associated with the progression and metastasis of multiple human cancer types. PDCD4 was first identified as a transformation suppressor gene in a mouse keratinocyte (JB6 cells) model of tumor promotion, in which high PDCD4 levels rendered cells resistant to transformation by the tumor-promoter 12-*O*-tetradecanoyl-phorbol-13-acetate (TPA) [6]. *PDCD4 *is known to play a role in apoptosis but its specific mechanism has yet to be determined. Recent studies indicate that *PDCD4 *may have important roles in transcription, translation, and signal transduction pathways (reviewed in [7]). *PDCD4 *has been suggested to function as a tumor suppressor, with reduced expression levels in cell lines derived from different tumor types [8-10]. *PDCD4 *levels were also decreased in primary patient tumor samples from lung cancer [8], hepatocellular carcinoma [10], breast carcinoma [11], colon cancer [12,13], glioma [14], pancreatic cancer [15] and esophageal carcinoma [16]. In epithelial tumors, such as breast cancer, PDCD4 protein expression levels were slightly reduced in ductal carcinoma *in situ*, but markedly decreased in invasive ductal carcinoma, suggesting that its loss may be required for invasion [17]. In colon carcinoma, PDCD4 over-expression was shown to decrease the invasive potential of cancer cells [18], and its under-expression enhanced cancer cell invasion [13].

microRNA (miR) target prediction databases suggest that *PDCD4 *is regulated by microRNA-21 (miR-21) [19,20]. Recently, *PDCD4 *has been shown to be regulated by miR-21 in other cancers, such as colon carcinoma [21]. It is known that microRNAs inhibit the expression of their target genes by degradation of target mRNA and/or translational repression of mRNA without degradation [22]. Interestingly, we recently showed that increasing miR-21 levels were significantly associated with progression of oral carcinoma lesions [23]. Binding of miR-21 to PDCD4 may thus be a potential mechanism of PDCD4 regulation in OSCC.

Recent experimental evidence suggests that PDCD4 function may be different according to cell type [7], thus highlighting the relevance of studying PDCD4 expression, its regulatory mechanism and its role in different cell and tissue types. Herein, we showed that *PDCD4 *levels were significantly decreased in OSCCs from node positive patients. We also demonstrated that PDCD4 is involved in invasion of oral carcinoma cells. Our data support a role for PDCD4 in OSCC invasion and metastasis. Further, we demonstrated that *PDCD4 *is regulated by miR-21 in OSCC. *PDCD4 *may have clinical utility for prediction, at the time of diagnosis, of which OSCCs may have a higher risk of neck nodal metastasis.

## Results

### QPCR results in relation to clinical data

**Figure 1:**
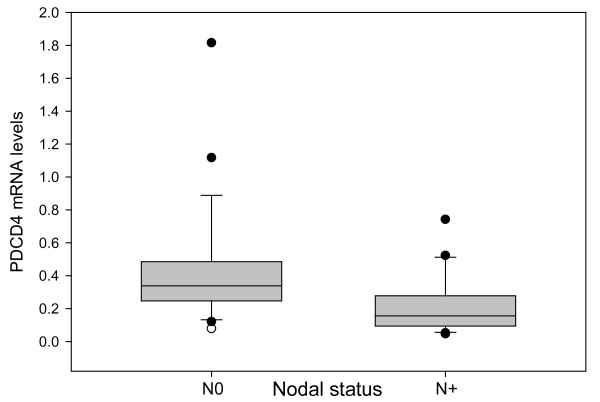
**Significantly lower PDCD4 mRNA levels were detected in OSCCs from node-positive patients (N+) as compared with node-negative patients (N0) (*p = 0.0027*)**. Median PDCD4 expression in node-positive tumors was 0.16 *versus *0.34 in node-negative tumors. The x-axis shows the nodal status (N0, N+), and the y-axis represents PDCD4 mRNA levels normalized against GAPDH and normal oral mucosa. In normal oral mucosa tissues (used as baseline control sample), PDCD4 expression levels were defined as 1, according to the Delta Delta Ct method of data analysis. Error bars represent standard error.

*PDCD4 *mRNA levels were decreased in 43/50 (86%) OSCCs, unchanged in six tumors and increased in one tumor (median, 0.28, range 0.05-2.82) compared to adjacent normal oral tissues (*PDCD4 *expression level in normal oral mucosa = 1). Lower *PDCD4 *mRNA levels were detected in OSCCs from patients with more advanced tumor stage (median, 0.25 *vs*. 0.35, *p = 0.054*), and were lower (median, 0.16 *vs*. 0.34; *p = 0.0027*) in tumors from patients with nodal metastasis (Figure 1). *PDCD4 *mRNA levels were not significantly different between other clinical variables.

### *PDCD4 *mRNA levels, survival and disease-free survival (DFS)

**Figure 2:**
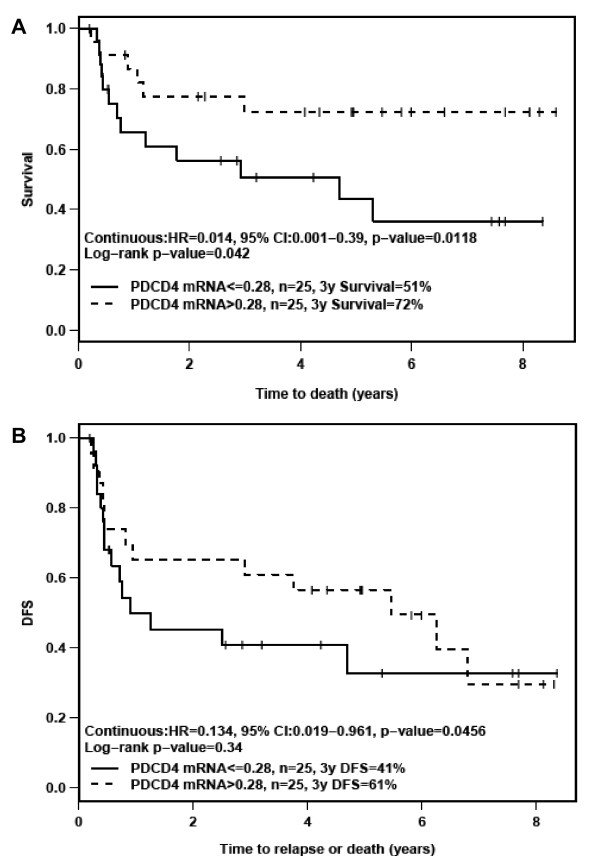
**A) Survival and B) Disease-Free Survival (DFS) of OSCC patients, according to PDCD4 mRNA levels**. DFS analysis showed 28 events (23 recurrences and 5 deaths without recurrence). Median PDCD4 expression at 0.28 was used as the cutoff point, which is regarded as unbiased.

Univariate analysis of survival and DFS showed that patients with lower *PDCD4 *mRNA levels showed worse survival (HR = 0.014, 95% CI: 0.001-0.39, *p = 0.0118*) and poorer DFS (HR = 0.134, 95% CI: 0.019-0.961, *p = 0.0456*) (Figure 2). Multivariate analysis including various clinical factors (e.g., perineural invasion, stage) yielded similar results; lower PDCD4 mRNA levels were associated with poorer survival (HR = 0.022, 95% CI: 0.001-0.713, *p = 0.0315*) and poorer DFS (HR = 0.12, 95% CI: 0.016-0.927, *p = 0.0421*). In the multivariate analysis, PDCD4 protein levels (as determined by IHC) were also associated with poorer DFS (HR = 0.564, 95% CI: 0.339-0.937, *p = 0.0272*). Although decreased PDCD4 mRNA and/or protein levels were associated with survival and/or DFS, these data are based on a small cohort of 50 patients.

### IHC results and correlation with clinical data

**Figure 3:**
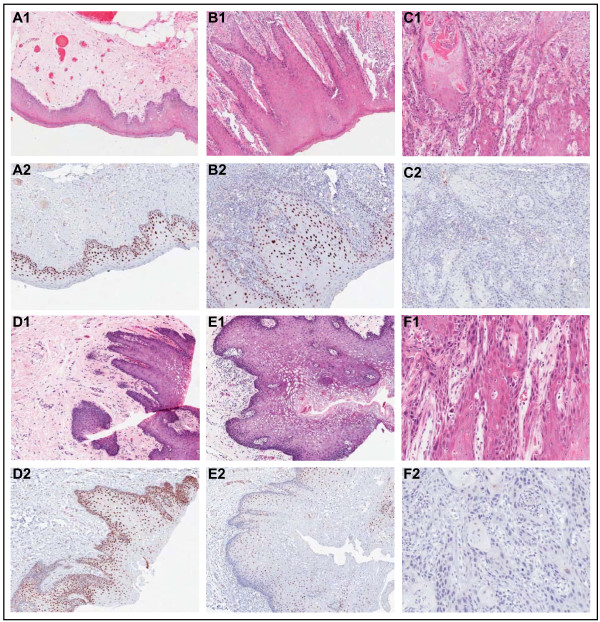
**Immunohistochemical analysis of PDCD4 shows the corresponding H&E-stained and PDCD4-stained tissue sections from patients with OSCC**. Panels A1, A2, D1, D2 show two adjacent normal epithelium samples with strongly positive, nuclear PDCD4 staining. Panels B1, B2, E1, E2 show two dysplasia samples with positive to weak nuclear PDCD4 staining. Panels C1, C2, F1, F2 show loss of PDCD4 expression in two moderately differentiated OSCCs. Normal, dysplasia and OSCC samples are paired and correspond to two different patients (A-C and D-F, respectively).

Immunohistochemical analysis showed that PDCD4 was strongly expressed in the nuclei of normal oral squamous epithelia (Figure 3, panels A and D). Nuclear PDCD4 staining was observed in dysplasia adjacent to the tumor, although with decreased intensity (Figure 3, panels B and E). PDCD4 expression was absent in the basal layer of normal and dysplasia samples. Overall, PDCD4 protein was absent/under-expressed in 25/28 (89%) of OSCCs (Figure 3, panels C and F), and marginally associated with nodal metastasis (*p = 0.059*). PDCD4 mRNA and protein levels were correlated (Wilcoxon signed rank, *p = 0.002*).

### PDCD4 expression in UT-SCC cell lines

**Figure 4:**
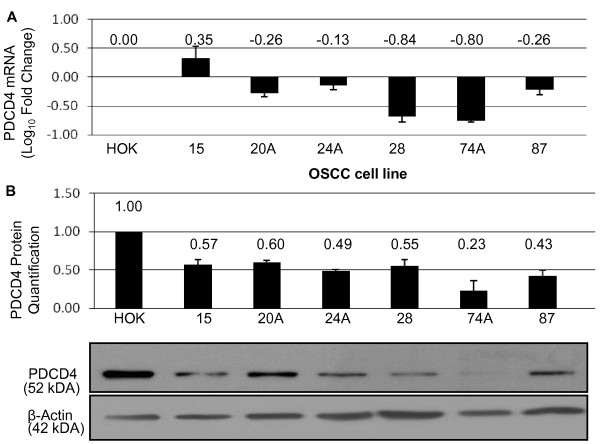
***PDCD4 *mRNA and PDCD4 protein levels in OSCC cell lines**. (A) The log_10 _ratio of *PDCD4 *mRNA in OSCC cell lines relative to HOK. (B) Quantification of PDCD4 protein expression in OSCC cell lines with a representative Western blot of PDCD4 protein in OSCC cell lines below. Cell line data are plotted mean ± SE and are representative of 3 separate experiments.

PDCD4 mRNA and protein expression levels were assessed in the UT-SCC cell lines. PDCD4 mRNA and protein levels were decreased in all cell lines, compared to the normal oral keratinocyte cell line (HOK) (Figure 4). UT-SCC-74A exhibited the lowest levels of PDCD4 mRNA and protein (Relative Ratio to HOK (RR) = 0.23), while UT-SCC-24A and 87 expressed similar levels of PDCD4 mRNA and protein levels (RR = 0.49, and 0.43, respectively).

### PDCD4 and tumor cell invasion

The optimal amount of transfected PDCD4 or PCMV6 control plasmids were tested by measuring the toxicity of various amounts of plasmid in UT-SCC-24A. We found that both 200 ng and 500 ng of PDCD4 plasmid led to an increase in PDCD4 mRNA and protein compared to the control. A plasmid concentration of > 500 ng was toxic to the cells (< 60% cell viability after 72 hrs) [see Additional file 1]. We then tested whether UT-SCC-24A would invade through a matrigel following transfection of 200 ng or 500 ng of PDCD4 compared to PCMV6; 200 ng of PDCD4 plasmid effectively decreased invasion compared to control (22 ± 6% *vs*. 80 ± 11%) and it was used for all subsequent experiments [see Additional file 2]. 500 ng PDCD4 and PCMV6 control plasmid also decreased invasion (1 ± 0% *vs*. 24 ± 2%).

**Figure 5:**
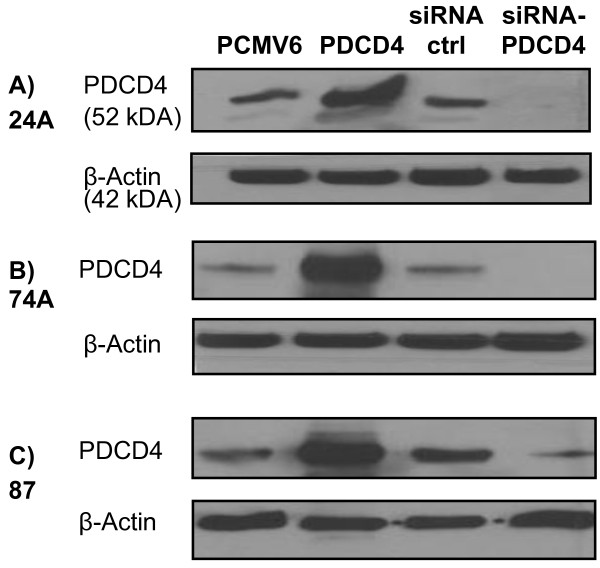
**Western blotting analysis demonstrating over-expression or knock-down of PDCD4 using PDCD4 plasmid or PDCD4 targeted siRNA, respectively, *versus *control plasmids (PCMV6, siRNA ctrl) in the UT-SCC cell lines (A) 24A, (B) 74A, and (C) 87**.

**Figure 6:**
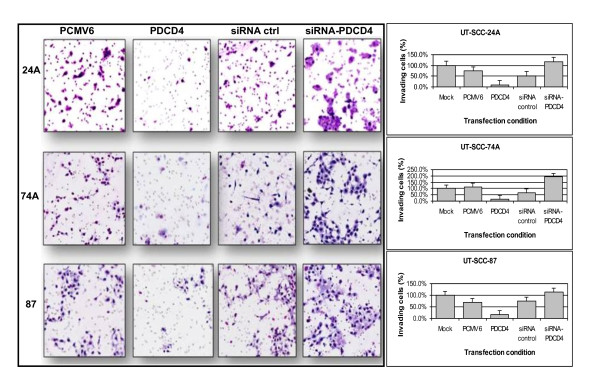
**Representation of invasion (5× magnification, left panel) following over-expression or knock-down of PDCD4 using PDCD4 plasmid or PDCD4 targeted si-RNA, respectively, *versus *control (PCMV6 and siRNA ctrl) in the UT-SCC cell lines 24A, 74A, and 87**. Cells were stained with the Diff-quick stain set. The percentage of invading cells (right panel) is shown for each cell line. Post-transfection, we observed a significantly decrease in the average percentage of invading cells, from 75% to 10% in UT-SCC-24A, from 113% to 17% in UT-SCC-74A, and from 69% to 16% in UT-SCC-87. Knock-down of PDCD4 resulted in an increase in the number of invading cells, from 52% to 118% in UT-SCC-24A, from 67% to 193% in UT-SCC-74A, and from 76% to 113% in UT-SCC-87.

Next, we determined the effect of over-expressing and silencing PDCD4 in UT-SCC-24A, 74A, and 87. Effective over-expression and knock-down of PDCD4 was confirmed by Western blot (Figure 5). Post-transfection, PDCD4 over-expression resulted in a significant decrease in the percentage of invading cells in all three cell lines, compared to mock-transfected control (Figure 6). Conversely, knock-down of PDCD4 significantly increased the number of invading cells in all cell lines, compared to mock-transfected control (Figure 6). These data suggested a role for PDCD4 in regulating OSCC invasion.

### miR-21 and PDCD4 in OSCC

**Figure 7:**
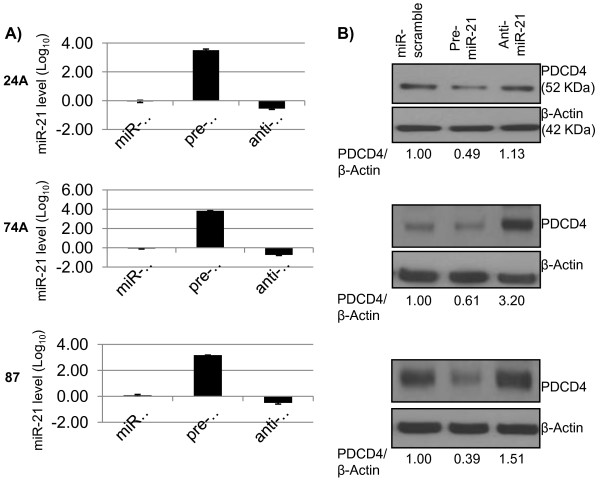
**Panel A shows miR-21 expression in pre-miR-21 or anti-miR-21 transfected cells compared to control (miR-scramble) in the UT-SCC cell lines 24A, 74A, and 87**. Panel B shows PDCD4 protein levels (Western blot) after transfection with pre-miR-21 or anti-miR-21 compared to miR-scramble control. PCR data plotted are the mean ± SE and are representative of 3 separate experiments. In the Western blot, PDCD4/β-Actin represents the ratio of the band intensity of PDCD4 compared to that of β-Actin, and are shown below the blots, for each cell line. Panels A-C in the same line corresponds to the same cell line, in this order (UT-SCC-24A, 74A and 87).

Since miR-21 is over-expressed and it has been shown to regulate PDCD4 in other cancers [21,24], we sought to determine whether miR-21 regulates PDCD4 in OSCC. First, we found that miR-21 was over-expressed (median, 12.79, range 2.78 - 63.67) in 28/28 primary patient OSCCs, as compared to normal tongue RNA (expression = 1). Next, we sought to investigate whether miR-21 regulated endogenous PDCD4 in UT-SCC cell lines, by examining the effect of transient transfection of miR-21 on PDCD4 protein levels. We transfected a range (25, 50, 75 or 100 pmol) of scramble-miR into UT-SCC-74A to determine the optimal plasmid concentration at 72 hrs. of transfection, without causing significant toxicity to the cells. We found that only 25 or 50 pmol of scramble-miR did not significantly decrease cell viability (91.3 ± 5.7% and 89.3 ± 2.4%, respectively) compared to mock-transfected. We tested the effect of transfection using 25 and 50 pmol of scramble-miR, pre-miR-21 and anti-miR-21 and both concentrations were confirmed as non-toxic to cells. By TaqMan real-time PCR, we confirmed that pre-miR-21 and anti-miR-21 led to over-expression and knock-down of miR-21, respectively. We found that pre-miR-21 successfully inhibited PDCD4 protein expression in all cell lines. Anti-miR-21 successfully up-regulated PDCD4 protein expression in UT-SCC-74A, and it only had a slight effect on PDCD4 expression in UT-SCC-24A and 87 (Figure 7). However, by site-directed mutagenesis (described below), we did show that miR-21 binds to the 3'UTR of PDCD4, causing its down-regulation.

### PDCD4 regulation by miR-21 in OSCC

**Figure 8:**
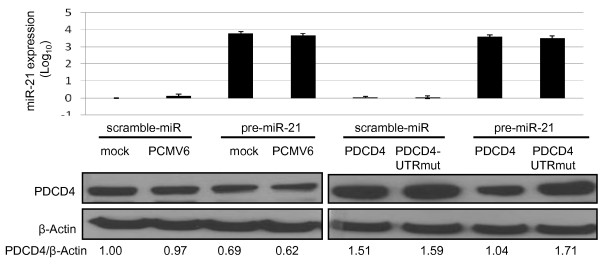
**The upper panel shows miR-21 expression levels following transfection with pre-miR21, PDCD4 and PDCD4-UTRmut, compared to controls: scramble miR and PCMV6 empty vector**. miR-21 expression data are presented as Log_10 _fold change, compared to mock-transfected control. Data are plotted as mean ± SE and are representative of two separate experiments. The lower panel shows the Western blot analysis of PDCD4 protein levels for the different transfection conditions. PDCD4/β-Actin represents the ratio of the band intensity of PDCD4 compared to that of β-Actin, and is shown below each blot. Co-transfection of miR-21 with PDCD4, but not PDCD4-UTRmut, resulted in a decrease in PDCD4 protein expression.

We showed that transient transfection of both PDCD4 and PDCD4-UTRmut led to up-regulation of PDCD4 compared to control. Co-transfection of miR-21 and PDCD4 resulted in a significant decrease in PDCD4 protein expression; however when miR-21 was co-transfected with PDCD4-UTRmut, PDCD4 protein levels remained unchanged (Figure 8). These results indicate that miR-21 directly binds to the PDCD4 3'UTR in oral carcinoma cells.

## Discussion

Herein, we identified PDCD4 under-expression at both the mRNA and protein levels in primary patient OSCCs and oral cancer cell lines. We showed that lower PDCD4 expression levels were significantly associated with regional disease (neck nodal metastasis), more advanced tumor stages, and poorer survival and disease-free survival of OSCC patients, suggesting that PDCD4 may have prognostic value in OSCC.

PDCD4 has been identified as a suppressor of tumorigenesis with lost or reduced expression in cancers of epithelial origin, including the lung [8], breast [11], colon [12,13], esophagus [16], and ovary [25]. In both lung [8] and ovarian [25] tumors, decreased or lost *PDCD4 *expression was associated with disease progression. In addition, a consistent decrease in PDCD4 expression levels was associated with the steps from normal to borderline to malignant ovarian tissues, and PDCD4 over-expression in ovarian cancer cells resulted in malignant growth inhibition [25]. In colon cancer, PDCD4 levels were continuously lower in the normal-adenoma-carcinoma sequence [12]. In other cancers, PDCD4 was shown to have diagnostic [12], and prognostic significance [8,26]. For example, loss of PDCD4 expression was correlated with poor patient prognosis, higher tumor grade and stage in lung cancers [8], shorter disease-free survival of ovarian cancer patients [25], as well as with clinicopathological features of tumor aggressiveness (e.g., nodal metastasis, advanced tumor stage) in gastric cancer [26]. PDCD4 was also shown to be an independent risk factor in colon [12,27] and lung cancer [8].

**Figure 9:**
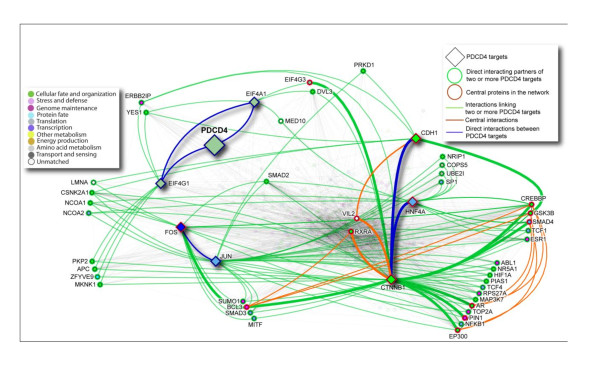
**Protein-protein interaction network of PDCD4 targets**. I2D version 1.72 was used to identify direct protein interactions for the PDCD4 targets (highlighted as diamond-shape nodes; n = 8). The resulting network of 523 proteins and 3,941 interactions was visualized using NAViGaTOR 2.1.14 http://ophid.utoronto.ca/navigator. Color of nodes corresponds to Gene Ontology biological function; shape of nodes represents different types of important proteins; color of edges represents different interactions among targets, as described in the legend. Blue edges signify direct interactions among PDCD4 targets. Green-highlighted nodes represent proteins interacting with at least two PDCD4 targets, and green edges identify corresponding interactions with PDCD4 targets. Red-highlighted nodes represent central proteins, and thick edges represent central interactions, and orange edges show direct interactions among central proteins and PDCD4 targets. Remaining nodes and edges were faded out to reduce network complexity. Network representation in XML file for NAViGaTOR, and annotation table, are provided in supplementary material.

Despite all of these studies showing PDCD4 deregulation associated with important clinical parameters in different cancers, the regulatory mechanisms of PDCD4 are still not completely understood. Recent research indicates that PDCD4 play roles in transcription, translation of proteins important in neoplastic transformation, such as eukaryotic initiation factors (eIFs) [28], as well cell signaling pathways; reviewed in [7]. PDCD4 regulates translation by interacting with the translation initiation factors eIF4A and eIF4G1 [29-31]. PDCD4 directly interacts with eIF4A via its MA3-c domain [28,32], inhibiting eIF4A helicase activity, and interrupting the assembly of the eIF4F complex [29,31,33], which results in disrupted cap-dependent translation and inhibition of cell transformation. Direct and indirect interacting partners of PDCD4 and its targets is shown in Figure 9, which was generated using I2D database [34] and NAViGaTOR network visualization system [35].

In our study, we showed that PDCD4 acted as a negative regulator of invasion in OSCC cell lines. Similar to our findings, PDCD4 suppressed the invasion and intravasation of colon cancer cells, implicating PDCD4 as regulator of invasion and metastasis [27]. Furthermore, PDCD4 down-regulation was shown to enhance invasion of colon cancer cells, by down-regulation, at least in part, of the transcription factor AP-1 components (c-Jun and c-Fos) [13]. PDCD4 was shown to act upstream of AP-1 to inhibit its activation. In addition, PDCD4 was shown to interact with c-Jun and to block its phosphorylation by JNK and prevent its recruitment of p300, a histone acetyl transferase required for transcription of AP-1 target genes by c-Jun [36]. These mechanisms lead to inhibition of c-Jun activity and down-regulation of AP-1 responsive promoters and a less invasive phenotype in colon cancer cell lines [18].

Other mechanistic studies in colon cancer showed that PDCD4 knock-down activates β-catenin/Tcf-dependent transcription and acts as a promoter of tumor cell invasion [13]. A recent study in PDCD4 knock-down colon cancer cell lines demonstrated that E-Cadherin loss is a key event for activating the β-catenin/Tcf-dependent transcription, leading to subsequent over-expression of the invasion-promoter genes u-PAR and c-MYC [37]. Although these studies demonstrated mechanisms of invasion regulated by PDCD4, the mechanisms that regulate PDCD4 in cancer cells are not well understood. Mechanisms that are frequently involved in the down-regulation of tumor suppressor genes commonly involve mutational inactivation and deletion [38], however such mechanisms seem not to apply to *PDCD4*. Several mechanisms of *PDCD4 *down-regulation were reported, such as hypermethylation of its 5' promoter region in glioma [39], increased proteasomal degradation [40], and silencing by miR-21 [21].

miR-21 has been shown to down-regulate PDCD4, leading to an increase in cell proliferation, invasion, metastasis, and neoplastic transformation of breast cancer lesions [15,41]. Additionally, miR-21 decreased PDCD4 levels and increased invasion and metastasis in colorectal cancer [27]. In esophageal carcinoma, down-regulation of miR-21 led to an increase in PDCD4 protein levels and a decrease in cellular proliferation and invasion [16]. We previously showed that miR-21 has continuously increasing over-expression during progression of oral carcinoma [23]. Previous studies have shown that PDCD4 is a putative target of miR-21 [21,41,42]. In our study, miR-21 over-expression in PDCD4-negative OSCCs suggested a potential mechanism of PDCD4 regulation by miR-21. We further demonstrated that miR-21 binds to the 3'UTR of PDCD4, causing its down-regulation. Our data thus suggest a mechanism of PDCD4 regulation by miR-21, leading to PDCD4 under-expression in oral carcinoma.

Considering that PDCD4 functions as a tumor suppressor gene and that it may have potential applications as a therapeutic target [43], understanding PDCD4 expression patterns, regulation, and role in OSCC may be valuable for exploring PDCD4 as a potential therapeutic target in OSCC.

## Conclusions

Our study showed significant PDCD4 under-expression/loss in malignant oral cavity tissues. Prominent loss of PDCD4 expression in metastatic OSCCs, correlated with poorer survival and poorer disease-free survival of OSCC patients suggests that PDCD4 may be a clinically relevant biomarker with prognostic value. PDCD4 loss may be one of the crucial steps required for invasion and metastasis of OSCC. In addition, our data also suggest that PDCD4 under-expression in OSCC may be regulated by miR-21.

## Materials and methods

### Patient Samples

This work was performed with the approval of the University Health Network Research Ethics Board. All patients signed informed consent before sample collection and were untreated before surgery. Tissue samples were obtained at time of surgery from the Toronto General Hospital, Canada. Patients are representative of the typical OSCC population within North America [44]. This study included a total of 50 patients used for gene expression analysis by quantitative RT-PCR (QPCR), and immunohistochemical analysis (IHC), as described below.

*QPCR: *QPCR analysis was performed in 50 primary patient OSCC samples. A subset of these patients (25/50) also had adjacent normal tissue (> 0.5 cm away from the tumor) collected. The detailed patient clinical data are described in Table 1. A head and neck pathologist (BP-O) performed histological evaluation of all samples to ensure that tumor specimens contained at least 80% of cancer cells and that all adjacent normal tissues did not have any evidence of dysplasia. Primary OSCCs and histologically normal samples were snap-frozen in liquid nitrogen until RNA extraction. Of these 50 patients, 28 patients had blocks available and were used for IHC analysis. Clinical data for this subset of 28 patients show clinicopathological characteristics representative of the original OSCC patient cohort [see Additional file 3].

**Table 1:** Clinical and histological characteristics of samples (QRT-PCR analysis - 50 patients)

Variables	N (%)
**Age (years)**	
Median (range)	67 (43-87)
**Gender**	
Male	36 (72)
Female	14 (28)
**Tobacco use**	
Yes	38 (76)
No	12 (24)
**Alcohol use**	
Yes	34 (68)
No	16 (32)
**Tumor Site**	
Tongue	33 (66)
Floor of mouth	10 (20)
Alveolar	4 (8)
Buccal mucosa	2 (4)
Retromolar	1 (2)
**T category**	
T1-T2	16 (32)
T3-T4	34 (68)
**Nodal status (pathological)**	
Negative (N0)	28 (56)
Positive (N1, N2b, N2c)	22 (44)
**Tumor Stage**	
I-II	14 (28)
III-IV	36 (72)
**Tumor Grade**	
Well differentiated	4 (8)
Moderately differentiated	41 (82)
Poorly differentiated	5 (10)
**Tumor thickness (mm)**	
Median (range)	12 (2-31)
**Perineural invasion**	
Yes	20 (40)
No	30 (60)
**Angiolymphatic invasion**	
Yes	16 (32)
No	34 (68)
**Recurrence**	
Yes	23 (46)
No	27 (54)
Outcome*	
Alive, no evidence of disease	25 (50)
Alive with disease	6 (12)
Dead of disease	15 (30)
Dead of other causes	4 (8)

* last date of follow-up: October, 2009.

### Cell Lines

Six OSCC cell lines (UT-SCC-15, 20A, 24A, 28, 74A, and 87), derived from primary patient samples, were supplied by Dr. Reidar Grénman, University of Turku, Finland [45]. Cell lines were maintained in Dulbecco's Modified Eagle Media (DMEM) containing 10% FBS, 1% Penicillin-Streptomycin and 1% L-Glutamine, at 37°C in a 5% CO_2 _humidified incubator. A normal oral mucosa cell line (Human Oral Keratinocyte, HOK, Invitrogen) was used as control. HOK cells were maintained in oral keratinocyte media, supplemented with 1% keratinocyte growth factor plus epithelial growth factor mixture (Invitrogen).

### RNA Isolation

Total RNA was extracted from patient samples (OSCC and normal tissues) and cell lines, using Trizol reagent (Life Technologies, Inc., Burlington, ON, Canada), followed by purification using the Qiagen RNeasy kit and treatment with the DNase RNase-free set (Qiagen, Valencia, CA, USA), all according to manufacturers' instructions. RNA was quantified using Nanodrop 1000 (Nanodrop); quality was assessed using the 2100 Bioanalyzer (Agilent Technologies, Canada). The RNA samples used were all of sufficient quality for gene expression analysis.

### QPCR Analysis

*PDCD4 *mRNA levels were examined in 50 OSCCs and 25 adjacent normal oral tissues, to verify whether *PDCD4 *was under-expressed in OSCCs and correlated with relevant clinicopathological data of patients. QPCR analysis was performed using the 7900 Sequence Detection System and the SYBR Green I fluorescent dye (Applied Biosystems, Foster City, CA) as previously described [5]. *GAPDH *was used as the internal control gene. Primer sequences were: *PDCD4 *Forward: 5'-ggcctccaaggagtaagacc-3'; Reverse: 5'-aggggtctacatggcaactg-3' and *GAPDH *Forward: 5'-aagggaaggttgctggatagg-3'; Reverse: 5'-cacatccacctcctccacatc-3'. Reactions were performed in triplicate for each sample and primer set. Dissociation curves were run for all reactions to ensure primer specificity and lack of PCR artifact. PCR products of randomly selected reactions were run on 1.5% agarose gels and visualized under UV, to verify presence of the appropriate-sized amplicon. *PDCD4 *mRNA levels in OSCC were normalized against 25 adjacent normal oral samples. Data were analyzed using the Delta Delta Ct method [5,46].

### Immunohistochemistry (IHC)

Tumor samples from 28/50 OSCC patients were analyzed by IHC. H&E-stained sections were examined for each patient. OSCCs included adjacent normal tissue in the same tissue section, which was used as the normal control. Tissue sections (4 μm) were cut from FFPE blocks and IHC staining was performed using the Avidin-Biotin method [47]. Sections were incubated with epitope-specific primary antibody against rabbit anti-human PDCD4 (600-401-964, Rockland Immunochemicals, Gilbertsville, PA, USA). For negative controls, antibody was omitted and antibody diluent alone or isotype matched IgG serum was used. Normal oral mucosa was used as a positive control.

### IHC Scoring Analysis

PDCD4 expression was evaluated semi-quantitatively [48], considering staining intensity (0 = absent; 1 = low; 2 = similar to normal epithelium; 3 = higher than normal) and percentage of positively stained cells (1 = immunostaining in ≤ 10% of cells; 2 = 11-30%; 3 = 31-60%; 4 = 61-100%). The final score was calculated by adding the intensity and percentage scores. Scores 1-4 represented absent/weak or under-expression and scores 5-7 represented no change or higher expression compared to adjacent normal. Slides were scored independently in a blinded fashion by three observers (B.P-O, PPR, and MT); the head and neck pathologist (B.P-O) examined all slides to ensure immunostaining specificity and quality.

### Statistical Analyses

Associations between *PDCD4 *mRNA expression and clinicopathological data were performed using the Rank-Sum test. The Fisher's exact test was used to correlate PDCD4 protein expression and clinical data. PDCD4 protein expression was dichotomized as negative (under-expressed) for scores 1-4 and positive (no-change or over-expressed) for scores 5-7.

For association with T category, tumors were grouped as T_1_-T_2 _*vs*. T_3_-T_4_, and for N category, tumors were grouped as node negative (N_0_) *vs*. node positive (N_1_, N_2 _and N_3_). Overall survival analysis was done using the Kaplan-Meier method. Survival was defined as the time between surgery date and death or last follow-up. Disease free survival (DFS) was defined as time between surgery date and recurrence or death or last follow-up. Statistical significance of differences between survival curves was assessed using Log-Rank test; Hazard Ratios (HR) and Confidence Intervals (CI) were calculated.

### Protein-protein interaction network

In order to determine functional relationships among the PDCD4 targets we mapped the 8 proteins (PDCD4, eIF4A, eIF4G1, JUN, c-FOS, CTNNB1, TCF (HNF4A), CDH1) to their corresponding SwissProt identifiers (SPIDs) [see Additional file 4]. All SPIDs were subsequently used to define a protein-protein interaction (PPI) network by querying Interologous Interaction Database (I2D; v1.72; http://ophid.utoronto.ca/i2d, with an update for BioGrid, DIP, HPRD, IntAct, MINT PPI data obtained 1/2010) [34]. PPI data for multiple SPIDs that map to the same gene (i.e., same Entrez Gene ID) were combined. The identified interacting proteins were then used to query the same database to determine whether any interactions are present between them to form the complete five prognostic signatures PPI network. PPI networks were visualized, annotated and analyzed using NAViGaTOR v2.1.14 http://ophid.utoronto.ca/navigator/[35].

### Plasmids

We used a commercially available PDCD4 expression plasmid, PCMV6-XL4-PDCD4 (Origene) and a control plasmid, PCMV6-XL4 (PCMV6; Origene). The PDCD4 plasmid consisted of the 2,640 base pair PDCD4 cDNA sequence (RefSeq: NM_014456) inserted into the PCMV6 multi-cloning site. *E. coli *cells were transformed with each plasmid and selectively expanded in ampicillin, using standard protocols. DNA was extracted using the Plasmid Midi-prep Kit (Qiagen) according to the manufacturer's protocol. DNA was quantified using Nanodrop 1000 (Nanodrop). In order to knock-down PDCD4 expression, we used a PDCD4 siRNA (50 μM) and control siRNA (50 μM) (Santa Cruz Biotechnology, CA, USA).

### Transfection

Cell lines were seeded in 6-well plates (2×10^5 ^cells/well) in DMEM containing 10% FBS, 1% Penicillin-Streptomycin and 1% L-Glutamine. Subsequently, cells were transiently transfected using previously described protocols [48], with either empty vector, PCMV6-XL4 (PCMV6; Origene), PCMV6-XL4-PDCD4 (PDCD4; Origene), 50 pmol of control si-RNA or PDCD4 si-RNA (Santa Cruz), 50 pmol of scramble-miR, pre-miR-21 or anti-miR-21 (Ambion) using Lipofectamine-2000 reagent (Invitrogen). Successful transfection without affecting cell viability was obtained for 3 cell lines (UT-SCC-24A, 74A, and 87). Transfection was confirmed by QPCR and Western blotting.

### Transwell Invasion Assay

We next carried out a transwell invasion assay to evaluate the invasive potential of 3 cell lines (UT-SCC-24A, 74A, and 87), which were successfully transfected with PDCD4, PDCD4 si-RNA or controls (mock, PCMV6 vector or si-scramble transfected). Transwell invasion assay experiments were carried out as previously described [48]. Cells that invaded the lower surface of the Matrigel-coated membrane were stained using the Diff Quick Stain set (Dade Behring, Newark, Del) and fixed onto a glass slide. The number of invading cells was quantified using NIH-ImageJ software [49] and normalized to mock-transfected controls. Data are representative of 3 independent experiments (biological replicates).

### Western Blotting

Samples were harvested from UT-SCC cell lines, total protein was extracted and protein concentration was determined using the Bradford Assay (Bio-Rad) as per manufacturer's instructions. Western blotting was performed using 25 μg of protein, according to standard procedures [48]. Immunodetection was done using anti-rabbit monoclonal antibody against PDCD4 (Rockland), diluted 1:5,000, followed by incubation with anti-rabbit secondary antibody (horseradish peroxidase HRP-conjugated) (GE Healthcare), diluted 1:5,000, for chemiluminescent detection. Anti-mouse monoclonal β-Actin (HRP-conjugated) (Santa Cruz), diluted 1:50,000 was used as control. The ECL plus detection system (GE Healthcare) was used, and signal intensities were determined by Image J software [49]. PDCD4 protein expression was determined semi-quantitatively based on ratio of the signal intensity of PDCD4 to β-Actin.

PDCD4 transfection did not affect cell viability, as verified by flow cytometry analysis using propidium iodide staining (Becton Dickinson FACS caliber, CellQuest software) (data not shown).

### *In Silico *Analysis for miR-21 Binding Sites

Since miR-21 is predicted to target PDCD4 [19,50,51], we sought to determine whether *PDCD4 *was a direct target of miR-21 in UT-SCC cell lines, or whether miR-21 was indirectly regulating PDCD4 by targeting another gene. For this, we first identified the sequence of potential miR-21 binding sites in the 3'UTR of *PDCD4*, using the online resource microRNA.org [20,52]. According to this analysis, miR-21 was predicted to bind *PDCD4 *with an alignment score of 157, based on the number of matching base pairs between the miR and its predicted binding site. Previous reports confirmed this potential miR-21 binding site at the 3'UTR of *PDCD4 *[41,53].

### Detection of miR-21 levels by TaqMan PCR

miR-21 levels were examined in the 28 patient OSCCs that had both PDCD4 mRNA and protein data available. PCR-based detection of mature miR-21 was performed using the TaqMan micro-RNA assays (Applied Biosystems). RT reactions were carried out using 100 ng total RNA by Multi-Scribe Reverse Transcriptase (50 units) in the presence of 1 mM dNTPs, 1× Reverse Transcription Buffer (Ambion) and RNase inhibitor (0.25 units) and miR-specific primers against the target sequences (miR-21, 5'-uagcuuaucagacugauguuga-3'; RNU44 endogenous control, 5'-ccuggaugaugauagcaaaaugcugacugaacaugaaggucuuaauuagcucuaacugacu-3'). miR-21 levels were normalized against RNU44 endogenous control miR [23], and calculated using the Delta Delta Ct method [5,46].

### Site-directed mutagenesis assay

The full length PDCD4 sequence (Origene) was used as a template to introduce mutations at the miR-21 binding site (PDCD4-UTRmut). This assay used the QuikChange Lightning Site-Directed Mutagenesis Kit (Stratagene). Briefly, PCR (18 cycles; annealing temperature of 68°C) was performed using PDCD4 as template and primers designed to introduce point mutations (bold); Forward: 5'-ggagggacagaaaagtaacctcttaagtggaata**ttctaagg**aattcccttttgtaagtgcc-3'; Reverse: 5'-ggcacttacaaaaggcccgggcttagaatattccacttaagaagaggttacttttctgtgtccctcc-3'. The PCR product was digested with the DpnI restriction enzyme, to remove any non-mutated DNA template. The digestion product was then transformed into competent DH5-alpha cells and plated onto ampicillin (50 ng/mL) coated agar plates; colonies were expanded and plasmid DNA was extracted using the Plasmid Mini-prep Kit (Qiagen). Sequencing analysis was performed and confirmed the presence of the PDCD4-UTRmut. PDCD4-UTRmut plasmid was then expanded in *E. coli *and plasmid DNA was extracted using the Plasmid Midi-prep Kit (Qiagen). 200 ng of PDCD4 or PDCD4-UTRmut plasmid were co-transfected with 50 pmol pre-miR-21 or scramble-miR (control) (Ambion). The pre-miR-21, anti-miR-21 and control miR (Ambion) were re-suspended in nuclease-free water to a concentration of 50 μM. Transfection experiments were performed in UT-SCC74A, as previously described [48]. RNA and protein were isolated after 72 hrs. Transfection of PDCD4 and pre-miR-21 was confirmed by Western blotting and QPCR, respectively.

## Competing interests

The authors declare that they have no competing interests.

## Authors' contributions

PPR and MT performed most of the work and contributed equally to this manuscript. PPR, MT, MS and SKR designed the study. PPR and MT performed analysis of patient samples. PPR and MS drafted the manuscript. MT conducted all functional experiments and edited the manuscript. NKC and JM performed part of the PCR experiments. JM contributed with study design. IJ and MP performed bioinformatics and statistical analyses, respectively. BP-O performed histopathological analysis of samples and scored the immunohistochemistry data. RG, PG and JI collected samples and helped collect clinical data. SKR supervised the study, helped with manuscript writing and approved the final manuscript. All authors read and approved the final manuscript.

## Supplementary Material

Additional file 1 **Cell viability of UT-SCC-24A after transfection with 200 ng, 500 ng, 1000 ng or 2000 ng of either PDCD4 or PCMV6 control plasmid compared to mock-transfected**. Click here for file

Additional file 2 **(A) Representation of invasion of UT-SCC-24A transfected with either transfection reagent alone (Lipofectamine-2000; mock), 200 ng or 500 ng PCMV6 or PDCD4 plasmid; (B) Quantification of invasion**. Data are plotted mean ± SE. Click here for file

Additional file 3 **Clinical details of the 28 patients used for PDCD4 IHC analysis**. These are a subset of patients shown in Table 1. Click here for file

Additional file 4 **PDCD4 network representation in XML file for NAViGaTOR, and annotation table**. Click here for file
