# Supplementary figures and images for: Programmed cell death 4 loss increases tumor cell invasion and is regulated by miR-21 in oral squamous cell carcinoma
Source: Mol Cancer. 2010 Sep 10;9:238. doi: 10.1186/1476-4598-9-238 (PMC2949797; doi:10.1186/1476-4598-9-238)

## Slide 1
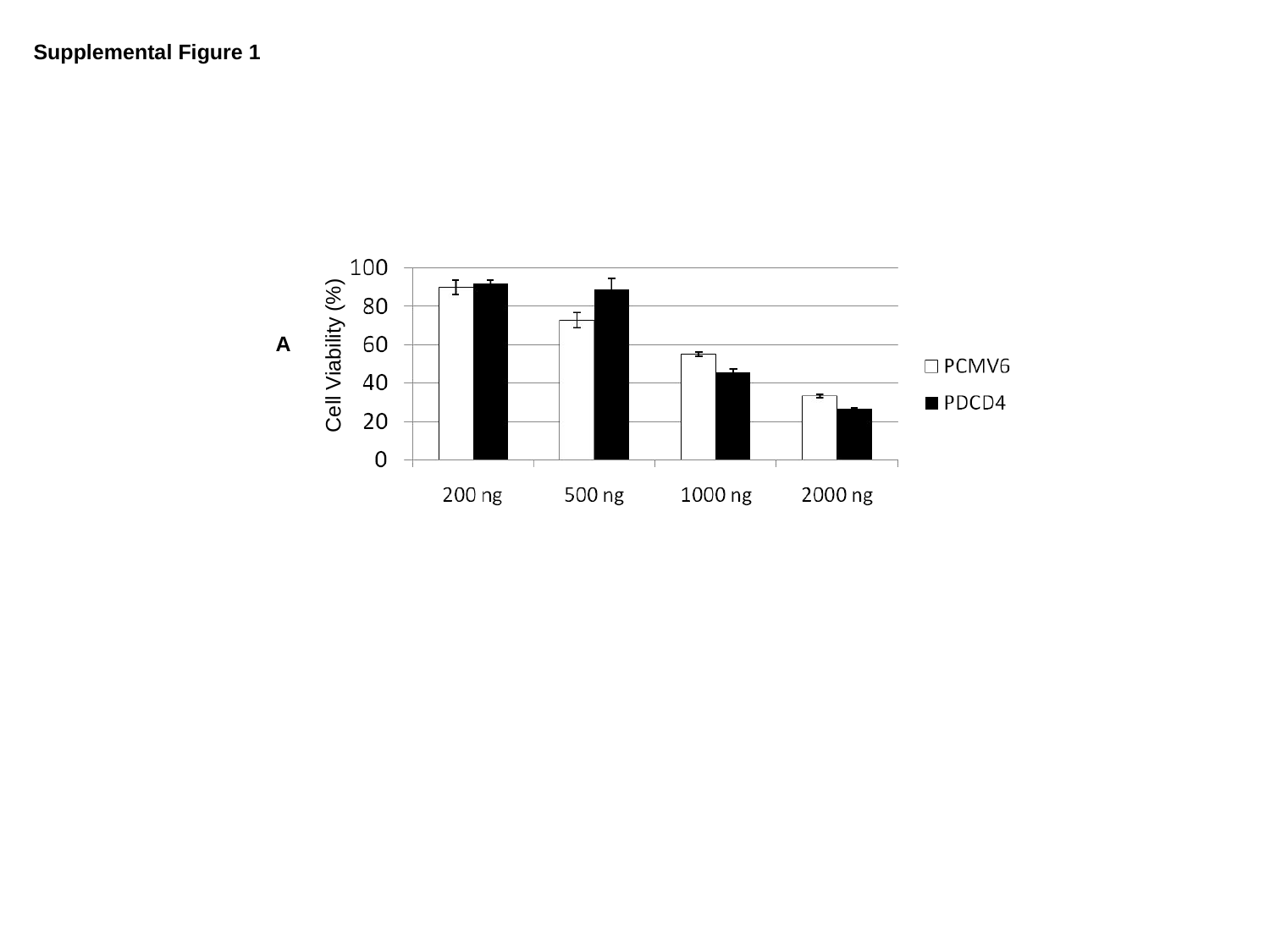

Supplemental Figure 1
A
Cell Viability (%)

Supplement: Additional file 1 — Cell viability of UT-SCC-24A after transfection with 200 ng, 500 ng, 1000 ng or 2000 ng of either PDCD4 or PCMV6 control plasmid compared to mock-transfected. [file 1476-4598-9-238-S1.PPT]
