# Supplementary material for: Programmed cell death 4 loss increases tumor cell invasion and is regulated by miR-21 in oral squamous cell carcinoma
Source: Mol Cancer. 2010 Sep 10;9:238. doi: 10.1186/1476-4598-9-238 (PMC2949797; doi:10.1186/1476-4598-9-238)
Supplement: Additional file 2 — (A) Representation of invasion of UT-SCC-24A transfected with either transfection reagent alone (Lipofectamine-2000; mock), 200 ng or 500 ng PCMV6 or PDCD4 plasmid; (B) Quantification of invasion. Data are plotted mean ± SE. [file 1476-4598-9-238-S2.PPT]

## Slide 1
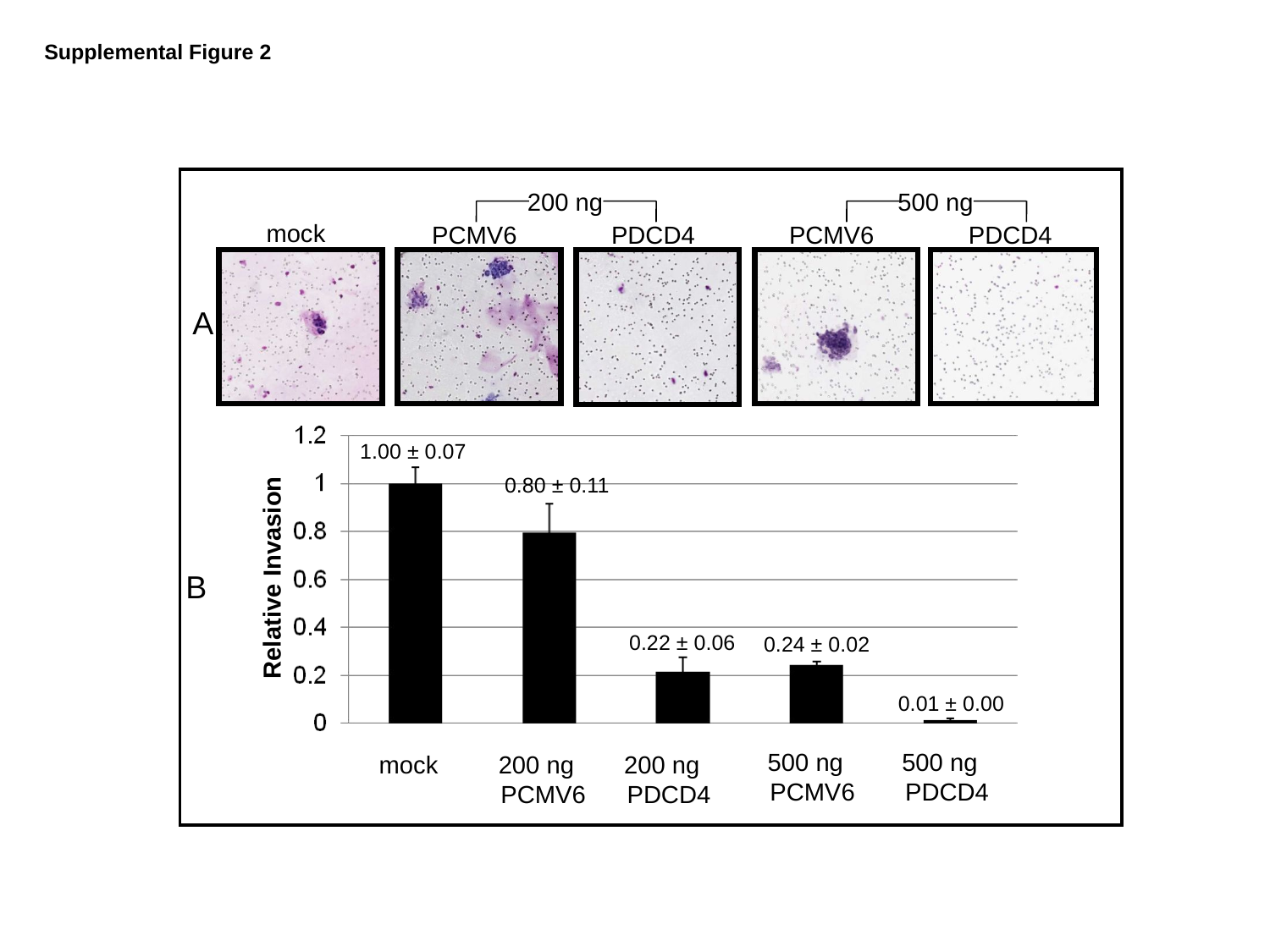

Supplemental Figure 2
200 ng
500 ng
mock
PCMV6
PDCD4
PCMV6
PDCD4
A
1.00 ± 0.07
0.80 ± 0.11
Relative Invasion
B
0.22 ± 0.06
0.24 ± 0.02
0.01 ± 0.00
500 ng PCMV6
500 ng PDCD4
200 ng PCMV6
mock
200 ng PDCD4
