# Supplementary material for: Programmed cell death 4 loss increases tumor cell invasion and is regulated by miR-21 in oral squamous cell carcinoma
Source: Mol Cancer. 2010 Sep 10;9:238. doi: 10.1186/1476-4598-9-238 (PMC2949797; doi:10.1186/1476-4598-9-238)
Supplement: Additional file 3 — Clinical details of the 28 patients used for PDCD4 IHC analysis. These are a subset of patients shown in Table 1. [file 1476-4598-9-238-S3.DOC]

**Supplemental Table 1.** Clinical details of the 28 patients used for PDCD4 IHC analysis. These are a subset of patients shown in Table 1.

| **Variables** | **N (%)** |
| --- | --- |
| Age (years) Median (range) | 67 (43-87) |
| Gender Male  Female | 20 (71.4)  8 (28.6) |
| Tobacco use Yes  No | 21 (75)  7 (25) |
| Alcohol use Yes  No | 18 (64)  10 (36) |
| Tumor Site Tongue  Floor of mouth  Alveolar  Buccal mucosa | 17 (61)  6 (21)  3 (11)  2 (7) |
| T category T_1_-T_2_  T_3_-T_4_ | 10 (36)  18 (64) |
| Nodal status (pathological) Negative (N_0_)  Positive (N_1_, N_2b_, N_2c_) | 16 (57)  12 (43) |
| Tumor Stage I-II  III-IV | 9 (32)  19 (68) |
| Tumor Grade Well differentiated  Moderately differentiated  Poorly differentiated | 2 (7)  24 (86)  2 (7) |
| Tumor thickness (mm) Median (range) | 12 (2-30) |
| Perineural invasion Yes  No | 10 (36)  18 (64) |
| Angiolymphatic invasion Yes  No | 7 (25)  11 (75) |
| Recurrence Yes  No | 10 (36)  18 (64) |
| Outcome* Alive, no evidence of disease  Alive with disease  Dead of disease  Dead of other causes | 15 (54)  3 (11)  6 (21)  4 (14) |
